# Humans and Other Animals—Future Friends or Foe?

**DOI:** 10.3390/ani2030361

**Published:** 2012-08-10

**Authors:** Clive J. C. Phillips

**Affiliations:** Center for Animal Welfare and Ethics, School of Veterinary Science, University of Queensland, Gatton Campus, Queensland 4343, Australia; E-Mail: c.phillips@uq.edu.au

Since the opening editorial, Animals has got off to an excellent start. Thirty articles were published in 2011, which comprise 446 pages of volume 1, and already 25 articles have been published this year in 360 pages of volume 2. Meanwhile the impact of other MDPI journals continues to grow; mean impact factor of the ten journals that have been in existence long enough to be assessed was 1.9, an increase of 0.3 from the previous year. Open access internet-based journals are proving ever popular and we eagerly await our first impact factor next year. There was a broad spread of topics, which can be roughly classified as follows (and the number of articles): greenhouse gas emissions from animals and climate change (10), animal ethics (9), veterinary medicine (8), animal nutrition (7), animal welfare (7), biodiversity (4) and animals in art and literature (4). Several book reviews have been published and replies by the authors. 

In the last editorial I asked the question—what would the planet be like without animals? A dramatic notion perhaps, but now I would like to suggest that it may one day be the fate of many of our most treasured animals unless we increase our research into this topic and present incontrovertible facts to the politicians demanding action. Animal habitat destruction is believed to be already widespread throughout the planet, as we make way for an ever expanding and demanding human population. Roads, houses, industry, shopping malls, cattle grazing—the erosion of the natural habitat of animals seems unstoppable. Large predators are particularly at risk as we deliberately remove them because of the potential danger to ourselves and our domestic animals. The biggest habitat destruction may yet result from anthropogenic climate change. Our farm animals are concentrated into larger and more intensive units for efficient use of labour and easy management. All this has come about in the last fifty years. The animal protection movement has gathered pace over this period, but is it adequate? What of the next fifty years? Will habitat destruction continue; will food production from animals really double to meet increased demand? Or will we find alternative ways of managing the food supply for an increasing population that don’t involve intensive animal keeping? Food scientists and agriculturists are trying to address the growing demand for food, but the impact on wild animals is often neglected. 

We must document declining numbers of animals in natural habitats, which often eventually leads to endangered species status. We must also document all of the effects that our manipulation of the environment is having on wild animals, from ever increasing numbers of road-kills to the impact of industrial activity on animal form and function. Several papers in the journal have addressed the impact of environmental change on animals. A new Special Issue with Guest Editor Professor John Koprowski will address the vitally important issue of declining biodiversity and the impact of habitat destruction. 

After the demise of many of our precious wild animal populations, what will happen to the captive animals that we have chosen to retain? Will we continue to intensify food animal production, often at the apparent expense of the animals’ quality of life? A proper understanding of the mental experience of our farm animals seems almost as elusive as it did at the start of animal welfare research about 50 years ago. Unless we have convincing evidence of emotional suffering, we cannot consider whether systems of farm animal management should be improved. And even if we have this evidence, what use is it unless we know the true relationship that we should be seeking with animals? Whilst cosmologists have made enormous strides in understanding the depths of our universe, we have done little to reveal the inner lives of managed animals much closer to home. The inaugural Special Issue from the Minding Animal conference of 2009 encouraged us to consider our future relationship with animals, and I am pleased to announce that the journal will publish a Special Issue dedicated to papers from the 2012 Minding Animals conference. 

The demise of animal populations to make way for an expanded human population might presage the demise of the human animal. Without other animals our potential purpose in the global ecosystem diminishes, no longer needed to manage animals, and deprived of the companionship, beauty, and enrichment of our lives that animals have provided for us in the past. 

